# Surface Level Ozone and its Adverse Effects on Crops and Forests: A Need for an Interdisciplinary Understanding

**DOI:** 10.1100/tsw.2001.24

**Published:** 2001-04-04

**Authors:** Sagar V. Krupa

**Affiliations:** Department of Plant pathology, University of Minnesota, St Paul 55108, USA

## Abstract

Surface level ozone (O_3_) is clearly a global scale problem with regard to its adverse effects on crops, forests and native, terrestrial plant ecosystems. Photochemists and meteorologists are continuing to define the chemistry and physics of the prevalence of O_3_ at the ground level. Similarly, plant scientists in the U.S. and Europe have examined the effects of O_3_ on crops and tree seedlings or saplings through large-scale studies. Examples include the U.S. National Crop Loss Assessment Network (NCLAN), the U.S. EPA’s (Environmental Protection Agency’s) San Bernardino National Forest Photochemical Oxidant Study, European Open-top Chambers Programme (EOTCP), and several ongoing EU (European Union) projects. In addition, there have been studies on mature tree responses through field measurements and by simulation modeling.

Virtually all of these efforts have attempted to relate air concentrations of $O_3$ to the observed plant responses. However, there is a rapidly growing awareness to link characteristics of atmospheric $O_3$ formation, its transport, flux onto leaf surfaces, plant uptake, and response. Clearly these processes are stochastic by their nature. Any satisfactory description of the cause and effect would require a close integration of knowledge from the multiple disciplines under consideration.

A recurring problem is the lack of satisfactory communication between atmospheric scientists, plant scientists, and cause-effect modelers. While frequently atmospheric scientists are not in touch with the finer aspects of biological processes, many biologists are not fully equipped to deal with the details from physical sciences. Thus, a clear and significant communication gap exists between the two groups. Some scientists continue to use integrated or average $O_3$ concentrations in geo-statistics and effects analyses. There are fundamental differences between such definitions and numerically defining the dynamics of the occurrences of ambient $O_3$ concentrations, their flux to the plant canopy and uptake, and the differing growth stages or time series in plant sensitivity and response. The net effect is governed by the products of a combination of stress and its avoidance or compensation and repair by plants.

Similarly, while human health effects scientists might consider multiple hourly running averages of $O_3$ levels in regard to air quality standards, plant effects scientists need to consider relationships based on time and conditions facilitating active uptake of $O_3$ into the leaves (the absorbed or effective dose) leading to an effect. Here, while breathing by humans is a continuous process, uptake of $O_3$ by leaves is a time-dependent diel process and is governed by factors such as global radiation, soil water availability, and presence of certain other pollutants.

There are several similarities between human cellular defense systems against $O_3$ and those in plants (e.g., presence of anti-oxidants such as ascorbate). Although the actual sequence of events leading to sensitivity or tolerance in plants to $O_3$ is not fully understood, ongoing studies with plant subjects can be complimentary to human research on oxidant stress[1].

There is no question that investigators specializing in physical sciences should not be expected to become experts in plant sciences or vice versa. However, there can be opportunities where basic information, in an understandable way, can be shared between specialists in multiple disciplines to gain a broader perspective.

A recent paper by Krupa et al. (2001)[2] provides a basic summary of the interdisciplinary aspects of the relationships between surface level $O_3$ and plant responses, and can prove very useful to non-plant scientists.
